# Metabolomics Approaches for the Diagnosis and Understanding of Kidney Diseases

**DOI:** 10.3390/metabo9020034

**Published:** 2019-02-14

**Authors:** Hayley Abbiss, Garth L. Maker, Robert D. Trengove

**Affiliations:** 1 School of Veterinary and Life Sciences, Murdoch University, 90 South Street, Perth 6150, Australia; g.maker@murdoch.edu.au; 2 Separation Science and Metabolomics Laboratory, Murdoch University, 90 South Street, Perth 6150, Australia; r.trengove@murdoch.edu.au; 3 Metabolomics Australia, Murdoch University Node, Murdoch University, 90 South Street, Perth 6150, Australia

**Keywords:** acute kidney injury, chronic kidney disease, diabetic nephropathy, kidney cancer, kidney disease, kidney transplantation, polycystic kidney disease, biomarker, chromatography, mass spectrometry, metabolomics

## Abstract

Diseases of the kidney are difficult to diagnose and treat. This review summarises the definition, cause, epidemiology and treatment of some of these diseases including chronic kidney disease, diabetic nephropathy, acute kidney injury, kidney cancer, kidney transplantation and polycystic kidney diseases. Numerous studies have adopted a metabolomics approach to uncover new small molecule biomarkers of kidney diseases to improve specificity and sensitivity of diagnosis and to uncover biochemical mechanisms that may elucidate the cause and progression of these diseases. This work includes a description of mass spectrometry-based metabolomics approaches, including some of the currently available tools, and emphasises findings from metabolomics studies of kidney diseases. We have included a varied selection of studies (disease, model, sample number, analytical platform) and focused on metabolites which were commonly reported as discriminating features between kidney disease and a control. These metabolites are likely to be robust indicators of kidney disease processes, and therefore potential biomarkers, warranting further investigation.

## 1. Introduction

The roles of the kidney include excretion, hormone production, regulation of blood pressure, and ionic, osmotic and pH balance [1,2], all of which make the kidney essential for physiological homeostasis [3,4]. Due to its many roles, diseases of the kidney often have systemic consequences making diagnosis and treatment extremely difficult and expensive. Kidney Health Australia, for example, estimates that the Australian government will be spending 7–8 billion dollars on the treatment of end-stage renal disease by 2020 [5]. Here we provide a description of some common nephropathies and their current status in terms of definition/diagnosis, aetiology, epidemiology and therapy/treatment. Also, the general workflow and available tools for conducting metabolomics experiments is provided. The purpose of this review is to assess a range of metabolomics studies since this approach can potentially determine metabolites of interest in kidney diseases, aiding the development of new diagnostic approaches and therapeutic targets for treatment. After providing a summary of kidney diseases and metabolomics, we examined a diverse selection of metabolomics studies of kidney diseases with the aim of highlighting metabolites which are consistently reported regardless of the disease model, study population size, sample type and/or analytical platforms used. Given that some overlap exists in the aetiology of some kidney diseases, these metabolites provide some insight into the biochemical processes linking different kidney diseases. 

## 2. Kidney Disease

**Figure 1:**
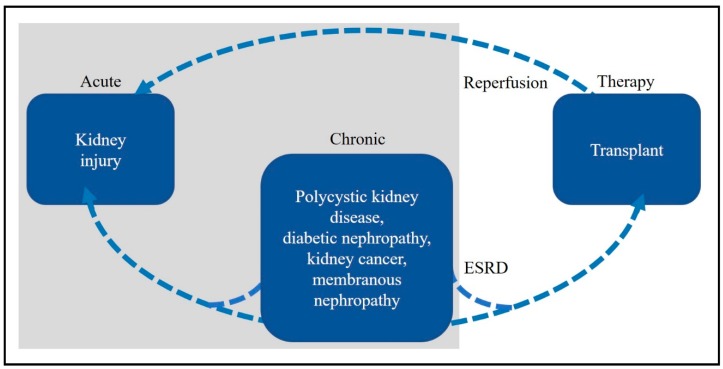
The overlapping relationships of the described kidney diseases. Chronic kidney disease and sub-entities: Polycystic kidney disease, diabetic nephropathy, kidney cancer and membranous nephropathy may progress to end-stage renal disease requiring transplantation. Reperfusion after transplantation may result in acute kidney injury. Acute kidney injury may also result from sub-entities of CKD.

There are many kidney diseases that are difficult to diagnose and treat despite a long history of studying the kidney and associated diseases. In his review, ‘Kidney’, Smith [6] cited literature from 1935, however it is known that the diagnoses of kidney disease dates back to at least the 1500s [7,8]. Treatments for chronic kidney disease are often invasive and expensive, and there are no known cures for chronic kidney diseases [5] or injuries [9]. Therefore, current research aims to develop targeted and effective diagnosis and treatment. We have included here a description of acute kidney injury (AKI) and chronic kidney disease (CKD) as they are often referred to as stand-alone conditions, and kidney transplantation, even though it is a therapy. In fact, AKI can be a result of, for example, reperfusion after transplantation, and conditions such as polyscystic kidney diseases (PKD), and diabetic nephropathy may result in CKD and the need for transplantation. Furthermore, AKI may develop in patients with CKD and vice versa [10]. These relationships are depicted in Figure 1.

### 2.1. Chronic Kidney Disease

CKD, the loss of kidney function over time (eGFR < 60 mL/min/1.73 m^2^ for three months or more [11]), affects 8–10% of individuals in Western Countries [12]. CKD has been classified into five stages according to glomerular filtration rate (GFR) or estimated GFR (eGFR; mL/min/1.73 m^2^). Creatinine-based estimations of GFR are most commonly used, however estimations based on cystatin C may also be used [13]. Table 1 lists the five stages of CKD [11] and further includes the classification of Stage 3 as 3a (45–59 mL/min/1.73 m^2^) or 3b (30–44 mL/min/1.73 m^2^). With no available cure for CKD, therapies centre on managing symptoms with renal replacement therapies such as dialysis and/or transplantation.

### 2.2. Diabetic Nephropathy

Diabetic nephropathy is the leading cause of chronic kidney disease, affecting approximately 30 to 40% of type 1 diabetes mellitus patients and 25 to 40% of type 2 diabetes patients [14,15,16,17]. Diabetic nephropathy is characterised by the combination of insulin- or non-insulin dependent diabetes with persistent microalbuminuria [17]. There is a genetic predisposition to diabetic nephropathy, however the most important risk factor is hyperglycaemia [17] and therefore management of diabetic nephropathy includes glycaemic control and pancreas transplantation to treat diabetes. It has been suggested that oxidative stress drives diabetic nephropathy through mitochondrial production of reactive oxygen species in response to hyperglycaemia [18]. Other factors contributing to progression include hyperlipidaemia, hypertension and proteinuria [17].

### 2.3. Acute Kidney Injury (AKI)

AKI is characterised by rapid loss of renal function [19] and may occur as a result of ischaemic or toxic damage to the kidney, both of which can also be caused by inflammation. AKI is diagnosed by increased serum creatinine and blood urea nitrogen (BUN; serum or plasma urea), estimation of glomerular filtration rate and urine output [19]. There are five stages of AKI for which patients are diagnosed based on the results: Risk, injury, failure, loss and end-stage renal disease (ESRD; RIFLE) [19,20] with loss and ESRD being outcomes of AKI rather than stages [9]. AKI is typically diagnosed after loss of renal function; therefore, risk factors have been identified and a number of early biomarkers which precede kidney damage have been proposed. Patient risk factors include sepsis, major surgery, bleeding and blood volume loss, and those with increased serum neutrophil gelatinase-associated lipocalin (NGAL), cystatin C and/or increased urinary NGAL, interleukin 18 (IL-18), kidney injury molecule 1 (KIM-1), glutathione S-transferase (GST) and liver-type fatty acid-binding protein (LFABP). All of these factors have been linked to kidney injury or disease [9,19,21,22,23,24,25,26] and have been recommended by Bellomo [19] as early biomarkers of AKI. 

### 2.4. Kidney Cancer

The incidence of renal cell carcinoma (RCC), the most common kidney cancer, is increasing [27]. The known subtypes of RCC are, in order of prevalence, clear cell (ccRCC), papillary (pRCC), chromophobe and collecting duct tumours [28]. In their review, Rini, Campbell and Escudier [29] estimated that the incidence of RCC is 209,000 new cases per year worldwide, with 102,000 deaths per year despite recent advances in drug therapies and surgical interventions. As well as genetic factors, the risk factors for developing RCC are smoking, obesity and hypertension [30]. Patients may present with local or systemic symptoms [29], however detection of RCC is usually early though incidental due to the recent widespread use of abdominal imaging. Stages 1 and 2 RCC outlined by Cohen and McGovern [30] are tumours less than (stage 1) or greater than (stage 2) 7 cm and limited only to the kidney. The 5-year survival for stages 1 and 2 is 95% and 88% respectively. Five-year survival declines to 59% and 20% for stages 3 and 4 respectively once tumours are beyond the kidney and lymph nodes are involved [30].

### 2.5. Kidney Transplantation

Kidney transplantation is a long-term treatment for chronic kidney diseases, however there are known risk factors associated with transplant rejection including acute rejection episodes, age, delayed graft function, diabetes, ethnicity and human leukocyte antigen (HLA) mismatching [31], with HLA matching the most robust predictor for transplant success [32]. Lee et al. [33] found that 100% of patients in their study developed human leukocyte antigen (HLA) antibodies prior to chronic rejection, however HLA antibodies also developed in patients without chronic rejection (27%). Detailed criteria for assessing histologic data from graft biopsy have been determined [34], as well as less invasive assessments. For example, in their 11-year study of over 100,000 kidney transplants, Hariharan et al. [31] found that serum creatinine (SCr) at 6 and 12 months post graft and delta SCr in the first year post graft would predict survival long-term.

### 2.6. Polycystic Kidney Diseases

PKDs are characterised by cyst formation, tubular defects and kidney enlargement and resultant dysfunction. There are at least two stages in the formation of cysts in PKD [35], the development and encapsulation of the cysts, and cyst growth [35]. It has been postulated that primary cilia are involved in PKD cystogenesis due to their role in the transport of cystoproteins (i.e., fibrocystin/polyductin) [36]. Renal cyst formation presents as a pathologic feature in Bardet-Beidl and Meckel-Gruber syndromes and the nephronophthisis (NPHP) group of ciliopathies [37], to which over 20 genes have been linked [38]. The most common presentation however is autosomal dominant polycystic kidney disease (ADPKD).

ADPKD has an incidence of approximately 1:400–1000 worldwide [39,40], making up 10% of adult patients with chronic renal failure requiring dialysis and/or transplantation [40]. ADPKD results in ESRD in 50% of patients by the age of 60 years [39,40] with hypertension, vascular aneurisms, cardiac valve defects and colonic diverticulae all associated with ADPKD due to the systemic nature of the disease [40]. ADPKD is caused by mutations in the Pkd1 (16p13.3) or Pkd2 (4q21–23) genes [41,42], however, there is evidence to suggest that a third locus exists [39,43]. Ariza et al. (1997) identified a Spanish family which presented with a mild form of ADPKD and showed negative linkage to the Pkd1 or Pkd2 loci. The phenotypes of the disease have been linked to the genotype, with the Pkd2 form resulting in a much milder form and presenting later in life than the Pkd1 form [39]. Although this pattern exists, variation within genotypes has been documented [40].

Autosomal recessive PKD (ARPKD) is less common, with an incidence of approximately 1 in 10,000–40,000, commonly evident in utero, with diagnosis by ultrasound at approximately 20 weeks [44,45,46,47] or at birth. Various clinical presentations have been observed in ARPKD [44], with the disease presenting as early as prenatally up to adulthood [41]. Arbeiter et al. (2008) postulated that, as with the ADPKD phenotypes, the severity of the ARPKD phenotype could not be explained by the genetic mutations [39] and more likely depends on other genes, epigenetic factors, environmental influences and potential modifier genes which have been identified in the described mouse models of PKD [48].

ARPKD is caused by mutations in the Pkhd1 gene on chromosome 6p12 [46] and is characterised by oligohydramnion, enlarged kidneys and lung hypoplasia [46]. The disease is also commonly characterised by fusiform dilation of the collecting ducts and distal tubules, as well as dysgenesis of the hepatic portal triad [46]. In up to 75% of severe cases, death is often caused by respiratory insufficiency and displacement of the diaphragm soon after birth. Neonates who do not exhibit pulmonary hyperplasia have a 50–80% survival rate. Of patients who survive the neonatal period, approximately 30% progress to end-stage kidney disease, commonly with hypertension and multiple organ failure [46].

In the past, depending upon the expressed phenotype of the disease, the management of ARPKD has been documented as aggressive [46,49]. Unilateral or bilateral nephrectomy has been described in four severe cases since 1993 [46,49]. It is of course likely that there are undocumented cases. In their case report, Arbeiter et al. (2008) described the treatment steps taken to alleviate the symptoms of ARPKD in a newborn female. ARPKD diagnosis was achieved using ultrasound and chest x-ray, and later confirmed histologically and genetically. Mechanical ventilation, administration of furosemide, hydrochlorothiazide, amlodipine, other diuretics and ultimately unilateral nephrectomy were included in the treatment strategy, however the patient died 27 days after birth. For presentations of ARPKD which are less severe, such as slow progression and presentation in later childhood or adulthood, treatment has still been described as invasive, i.e., renal replacement therapy.

There is currently no cure available for PKD [36], however with recent approaches in understanding the molecular pathogenesis and cystogenesis of the disease, such as targeting mTOR activity [50], EGFR axis [46], and cAMP-activated B-Raf/ERK [51,52] and (IRS) PI3K/Akt [53] signalling pathways, new therapies and management strategies are emerging and advancing into clinical trials [36]. In recent preclinical models of PKD, Bukanov et al. [54] and Natoli et al. [55] have shown that inhibition of dysregulated cell cycle and apoptosis with the CDK inhibitor, roscovitine, leads to arrest of PKD.

## 3. Metabolomics

Downstream of genomics, transcriptomics and proteomics, metabolomics is the comprehensive study of metabolites in the cell, tissue and/or biofluids, collectively known as the metabolome, of an organism [56]. A metabolite is typically defined as a small molecule intermediate or product of metabolism of molecular weight less than 1500 Daltons [57]. Metabolism is a close representation of the phenotype [58], and with its capacity to represent the function of an organism as a result of a disease or environmental stress at a particular time [59], it is not surprising that metabolomic approaches have been gaining popularity. 

A comprehensive database of the human metabolome has been compiled (The Human Metabolome Database; HMDB; [60,61,62,63]) including over 100,000 known, expected and predicted metabolites with compound origin, biofluid/tissue location, spectra and reference ranges if available. The HMDB classifies metabolites by super class, class and sub class with sub class including well known metabolites classes such as amino acids, carbohydrates, and fatty acids.

Metabolomics aims to detect both endogenous and exogenous metabolites including small molecules taken up from food, medicines and the environment. There are two key approaches in metabolomic experiments, termed targeted and untargeted, although there are many terminologies for these approaches in the literature [64,65]. Targeted experiments are conducted when pre-defined metabolites are of interest and their concentrations (relative, semi-quantitative or absolute) are subsequently measured. Untargeted approaches are conducted when there is no prior knowledge of the metabolites being measured and the concentrations (relative or semi-quantitative) of as many metabolites as possible are determined. In the context of this review, both approaches may determine metabolites which differentiate between, for example, individuals with kidney disease and healthy individuals, though novel markers are more likely to be uncovered using an untargeted approach. 

The analytical platforms with which metabolomic experiments are conducted are predominantly nuclear magnetic resonance (NMR) spectroscopy and chromatography-mass spectrometry (MS), however other techniques have been described [66] and complementary platforms are desirable for greater metabolome coverage [67]. NMR spectroscopy provides a quantitative, robust platform that requires larger volumes of sample, but minimal sample preparation. MS provides sensitivity which ultimately gives a greater metabolome coverage within a single injection, but is less robust and requires detailed sample preparation, though smaller sample volumes may be used. We focus on mass spectrometry approaches in further detail in the following sections however findings from NMR-based studies are included in the final section. 

### 3.1. Sample Collection, Preparation, Storage and Handling

The selection of an appropriate sample to study and the way in which it is collected, prepared, stored and handled will, in combination with extraction, analytical platform, data analysis and metabolite identification, be key to the interpretation of results. A review by Walsh et al. [68] identified the challenges in metabolomic research regarding each stage of a metabolomic experiment, but with a particular focus on sample collection, preparation storage and handling. [69] The impacts of such pre-analytical procedures on plasma and serum metabolomes have recently been reviewed in detail by Yin, Lehmann and Xu [70]. As an example, an endogenous metabolite was found in varying levels as a contaminant in different plasma and serum vacutainer tubes [69] which highlights the importance of keeping protocols consistent. There are still no agreed methods for the collection, preparation, storage and handling of samples for a metabolomic experiment [71,72], however the Metabolomics Standards Initiative (MSI) [73] have recommended reporting criteria for mammalian/in vivo biological samples [74]. 

In addition to experimental samples for MS-based untargeted metabolomic experiments, it is now widely accepted that pooled quality control samples providing technical replication throughout an analytical sequence and batches of sequences should be used [75]. Similarly, reference samples or test mixtures have been proposed to assess analytical performance [76,77]. For the subtraction of artefacts introduced through sample collection, preparation, storage, handling, extraction and analysis, ‘method’ or ‘blank’ samples should also be processed throughout these stages.

### 3.2. Metabolite Extraction

The physicochemical properties of metabolites vary greatly and therefore cannot be extracted with a single solvent, nor analysed on a single analytical platform [78]. Many studies report adaptations of the Bligh and Dyer lipid extraction method [79] to utilise both polar and non-polar fractions. Metabolite extraction methods include steps for protein precipitation, metabolite extraction (usually a single step for both) and filtration or other form of sample clean-up such as solid phase extraction (SPE) [65]. Commonly reported solvents for the precipitation of protein and metabolite extraction are acetonitrile, methanol and water, though acidification and heating have also been described.

### 3.3. Chromatographic Separation

Chromatographic separation of analytes prior to detection reduces the complexity of spectra for sample matrices by reducing interference from neighbouring peaks and aids in the identification of metabolites by providing retention time (and/or index) information for a compound. It is not always possible however to resolve all peaks, particularly in untargeted analyses. Chromatography coupled to MS has been used extensively in metabolomic research [80], the most common platforms being gas chromatography (GC) and liquid chromatography (LC).

#### 3.3.1. Gas Chromatography

Gas chromatography has been widely used in metabolomics-based research [81] and has demonstrated good metabolome coverage (i.e., detection of a variety of metabolite physicochemical properties), as well as detecting unique compounds in the human serum and urine metabolomes [82,83]. Gas chromatography is ideal for low molecular weight volatile compounds and in metabolomic experiments, provides superior chromatographic resolution and retention time reproducibility when compared to liquid chromatography. To give greater metabolome coverage, however, some metabolites must first be derivatised. Derivatization reduces polarity, increases thermal stability and improves resolution and peak shape/intensity [84,85]. Derivatisation can be cumbersome and artefacts and multiple derivatisation products can be observed, however there has been good uniformity in GC-MS based methods allowing the curation of extensive databases for the identification of metabolites analysed by GC-MS, such as the National Institute of Standards and Technology (NIST) Mass Spectral Database. Examples of public libraries for the identification of GC-MS spectra include Golm Metabolome Database [86] and FiehnLib mass spectral library [87]. 

#### 3.3.2. Liquid Chromatography

Liquid chromatography approaches can capture a wider range of metabolite physicochemical properties than GC-MS and have generally yielded more sensitive acquisitions and hence greater numbers of metabolites. However, a wide range of column chemistries have been employed, as well as a wide range of mobile phase solvent preparations, resulting in different retention times and adduct formations, decreasing confidence in spectral matching with commercially and publicly available spectral libraries.

### 3.4. Mass Spectrometry

#### 3.4.1. Ionisation

For GC-MS platforms, ionisation is achieved most commonly by electron ionisation (EI) using 70 eV, however chemical ionisation using reagent gases such as methane and ammonia may be used. EI is a hard ionisation technique causing considerable fragmentation of compounds. EI fragmentation rarely allows detection of intact molecular masses, however the fragmentation patterns are highly reproducible, allowing confident identification of features. Compounds with similar structures show similar fragmentation patterns and are more difficult to identify without specialised chromatographic methods preceding MS detection. Electrospray ionisation is most commonly used in LC-MS applications and is a ‘soft’ ionisation technique, allowing the detection of intact compounds and measurement of pseudo-molecular masses. ESI is less reproducible and depends heavily on the specific mass analyser and manufacturer [65]. 

#### 3.4.2. Mass Analysers

The most commonly employed mass analysers in metabolomics are quadrupole (Q), time-of-flight (TOF) and ion trap (IT)/orbitrap, with tandem variations including triple quadrupole (QQQ), QTOF, TOF/TOF, IT/TOF. Single and triple quadrupole systems generally provide unit mass resolution and, in the case of the QQQ, high sensitivity. Time-of-flight instruments provide high resolution, accurate mass data, as does the orbitrap. Kind et al. [88] have recently outlined common MS platforms and their performance characteristics. 

### 3.5. Data Processing and Analysis

Chromatography-mass spectrometry metabolomic data requires feature (mass-to-charge peak) detection, deconvolution and retention time alignment. There are many software packages and computational tools for this, both commercial and public, with one of the most commonly reported peak picking and retention time alignment tools being XCMS [89] for LC-MS workflows. Deconvolution is essentially the grouping of *m/z* ions of a single component and is essential for GC-EI-MS data [90]. Similar approaches are needed for LC-MS data where multiple adducts are present. We previously mentioned quality control measures including pooled samples, reference samples and test mixtures. At this point these should be utilised to determine the quality of data and remove features/samples which are irreproducible including those which appear to be, for example, sample mismatches or extreme values. Various forms of quality control measures for metabolomics studies have recently been reviewed [91]. Statistical analyses are then conducted to prioritise identification and interpretation of features from untargeted metabolomics experiments. Prior to statistical analyses, centring, scaling or transformation of the data are carried out [92]. Tools such as PhenoMeNal [93], Metabolomics Workbench [94] and MetaboAnalyst [95,96,97,98,99,100] offer data analysis solutions.

### 3.6. Metabolite Identification and Interpretation of Findings

#### 3.6.1. Identification

For targeted experiments, metabolite identification is considered in the early stages of the chosen data analysis pipeline, but for untargeted approaches, it is usually the final stage of data processing, occurring after metabolites of interest have been determined. For targeted experiments, authentic reference standards are purchased and analysed ahead of the experiment. In untargeted experiments, commercial and public spectral libraries are used, as well as online databases to match and putatively identify MS, as well as MS/MS and MS^n^ experimental spectra [88]. These identifications may be supported by purchasing the authentic reference standard, or laboratories may have in-house platform-specific spectral libraries for confirmation of metabolite identifications. 

Reporting the confidence of metabolite identifications in metabolomic experiments has recently been addressed in the literature [101,102]. Initially, levels of identification were proposed [80] where, as described by Sumner et al. [80], a Level 1 identification is confirmed with an authentic standard of the compound and Level 4 is an unidentified compound. Schymanski et al. [103] described five identification levels for high resolution data where, similar to Sumner et al., [80], Level 1 is confirmed with an authentic standard. Level 4 is unidentified, but has an unequivocal molecular formula and Level 5 is a ‘mass of interest’. More recently, Sumner et al. [102] proposed alphanumeric scoring metrics for metabolite identification in order to communicate the confidence in an identification.

#### 3.6.2. Interpretation

The biological interpretation of data relies first on the identification of significant metabolites and second on mapping those metabolites to biochemical pathways and validating these data with other sources of data such as, for example, HMDB [60,61,62,63], GWAS Catalog [104], SNiPA [105], PhenoScanner [106] and www.metabolomix.com. Examples of currently available resources for mapping metabolites to biochemical pathways include the BioCyc database collection [107], KEGG pathway database [108], MetaboAnalyst [95,96,97,98,99,100,109], the Small Molecule Pathway Database (SMPDb; [110,111]) and Recon3D [112]. 

## 4. Findings from Metabolomic Studies of Kidney Disease

Metabolomics in the study of kidney disease has been reviewed over the past five years [8,12,113,114,115,116,117,118,119], elegantly summarising the application of metabolomics to kidney disease and the recent findings of such studies. A selection of recent metabolomic studies of kidney disease has been included here (Table 2), providing the disease, model, lowest recorded *n* per sample group, sample type and platform on which the metabolomic data was acquired. Metabolomic-based kidney disease studies have been carried out using mouse and rat models, but the majority of studies listed here have used human participants. Many of the studies presented in Table 2 reported relatively low sample numbers. For studies using animal models where experimental conditions are highly controlled, this may be less of an issue. For studies using human participants, however, especially for CKD where the cause of kidney disease may be variable, this issue has started to be addressed with eight studies since 2015 reporting >50 subjects per group. Indeed, two of these studies reported sample numbers approaching 1000 per group. Whether urine, plasma, serum or kidney tissue were used, many of the same markers have been found. For example, hippuric and uric acids have been shown to discriminate kidney disease based on urine [120], serum [121] and kidney tissue [122]. Moreover, uric acid has been detected using both GC- [120] and LC-MS [121]. Hippuric and uric acids have been found as markers for both CKD [121] and PKD [120]. 

We have included a range of sample numbers, types, and analytical platforms to begin to assess overlapping biochemical processes. Table 3 shows metabolites which have been reported as important for two or more of the seven kidney diseases listed in Table 2. Although this list is only a snapshot of the currently available literature, it provides biochemical insight into some of the common processes linking kidney diseases. With allantoin and quinolinic acid each found in four of seven kidney diseases we propose that purine metabolism and tryptophan metabolism via the kynurenine pathway are important for kidney diseases.

### 4.1. Purine Metabolism

Allantoin is a product of purine metabolism excreted in the urine of most mammals except in humans, where uric acid is usually excreted [63], unless it is first oxidised to allantoin [155]. Allantoin was reported in studies of CKD, diabetic nephropathy, PKD and kidney transplantation (Table 3) and uric acid in CKD, diabetic nephropathy and PKD. Xanthosine and hypoxanthine were reported in CKD and transplantation, and CKD and PKD respectively, further supporting the role of purine metabolism in kidney disease. In humans, allantoin has been proposed as a marker of oxidative stress [155], however in this collection of studies, allantoin was only found in rat models of disease [120,122,129,139,147,152]. Two studies of CKD using human participants reported uric acid [121,126], the synthesis of which has been directly linked to oxidative stress [156] through the conversion of xanthine dehydrogenase to xanthine oxidase, a known source of reactive species [156]. Uric acid as a uremic toxin/retention solute may also be a source of oxidative stress [156].

### 4.2. Tryptophan Metabolism

Quinolinic acid is synthesized from tryptophan via the kynurenine pathway [157]. This metabolite, reported for CKD, transplant, membranous nephropathy and kidney cancer, as well as the metabolites kynurenic acid, tryptophan and indoleacetic acid, suggests that tryptophan metabolism plays a role in kidney diseases. The kynurenine pathway has previously been associated with oxidative stress and inflammation in ESRD patients [158]. Furthermore, it has been demonstrated that the ratio between plasma kynurenic acid and tryptophan is a robust indicator of kidney function [159] and that there may be involvement of the tryptophan pathway in chronic kidney disease possibly via increased activity of the enzymes kynurenine aminotransferase (KAT), indoleamine 2,3-dioxygenase (IDO) in the kidney and/or tryptophan 2,3-dioxygenase (TDO) in the liver.

## 5. Conclusions

Metabolomics studies have provided many candidate small molecules which may be appropriate as biomarkers for kidney disease and also for elucidating mechanisms in disease onset and progression. Here we have determined that both purine metabolism and tryptophan metabolism are likely to play key roles in kidney disease since allantoin and quinolinic acid have been consistently reported, as have other metabolites of their respective biochemical pathways. These metabolites and pathways have previously been linked with oxidative stress and inflammation which are known complications of kidney disease and may contribute to the progression of chronic kidney disease to ESRD. These interpretations are limited however to the mapping of identified metabolites to pathways, while many important features remain unidentified. As databases grow, these data can be further interrogated to understand kidney diseases.

## Figures and Tables

**Table 1:** Stages of chronic kidney disease as defined by glomerular filtration rate (GFR) or estimated glomerular filtration rate (eGFR).

Stage	GFR or eGFR (mL/min/1.73 m^2^)
1	≥ 90
2	60–89
3	30–59
3a	45–59
3b	30–44
4	15–29
5	<15

**Table 2:** Selection of metabolomic studies of kidney disease. *n* = minimum number of samples in a study group reported for any sample type.

Kidney Disease	Reference	Model	*n*	Sample Type	Analytical Platform
Acute kidney injury	Sun, 2012 [123]	Human	17	Serum	LC-MS
Chronic kidney disease	Shah, 2013 [124]	Human	10	Plasma	GC-MS, LC-MS
	Zhao, 2013 [122]	Rat	8	Kidney tissue	LC-MS
	Boelaert, 2014 [121]	Human	20	Serum	GC-MS, LC-MS
	Luck, 2016 [125]	Human	110	Urine	NMR
	Rhee, 2016 [126]	Human	200	Plasma	LC-MS
	Sekula, 2016 [127]	Human	991	Serum	GC-MS, LC-MS
	Kobayashi, 2015 [128]	Human	69	Plasma	LC-MS
	Zhao, 2013 [129]	Rat	12	Kidney tissue	LC-MS
	Atzori, 2011 [130]	Human	13	Urine	NMR
	Nkuipou-Kenfack, 2014 [131]	Human	10	Plasma, urine	LC-MS
	Mutsaers, 2013 [132]	Human	≥4	Plasma	NMR
	Zhang, 2015 [133]	Rat	8	Urine	LC-MS
	Qi, 2012 [134]	Human	20	Serum	NMR
	Zhao, 2013 [135]	Rat	8	Serum	LC-MS
	Goek, 2013 [136]	Human	87	Serum	LC-MS, FIA-MS
Diabetic nephropathy	Van der Kloet, 2012 [15]	Human	26	Urine	GC-MS, LC-MS
	Stec, 2015 [137]	Mouse	11	Urine	NMR
	Sharma, 2013 [138]	Human	12	Plasma, urine	GC-MS
	Zhao, 2012 [139]	Rat	12	Kidney tissue	GC-MS, LC-MS
	You, 2015 [140]	Mouse	11	Urine	GC-MS
	Makinen, 2012 [141]	Human	86	Serum	NMR
	Makinen, 2008 [142]	Human	137	Serum	NMR
	Makinen, 2013 [143]	Human	63	Serum	NMR
	Barrios, 2018 [144]	Human	926	Serum	NMR
Kidney cancer	Kind, 2007 [145]	Human	6	Urine	GC-MS, LC-MS
	Kim, 2011 [146]	Human	11	Urine	LC-MS
Kidney transplantation	Serkova, 2005 [147]	Rat	6	Kidney tissue, whole blood	NMR
	Stenlund, 2009 [148]	Human	19	Urine	NMR
	Suhre, 2016 [149]	Human	241	Urine, kidney tissue	GC-MS, LC-MS
Membranous nephropathy	Gao, 2012 [150]	Human	14	Serum, urine	GC-MS
Polycystic kidney disease	Taylor, 2010 [151]	Mouse	9	Urine	GC-MS
	Toyohara, 2011 [152]	Mouse	5	Plasma	CE-MS
	Abbiss, 2012 [120]	Rat	6	Urine	GC-MS
	Gronwald, 2011 [153]	Human	10	Urine	NMR
	Hwang, 2015 [154]	Mouse, human	2	Cells, plasma, tissue, urine	GC-MS, LC-MS

**Table 3:** Metabolites which have been reported as important for two or more kidney diseases from the list of studies in Table 2. Darker colour indicates metabolite reported in greater number of kidney diseases. CKD: Chronic kidney disease; DN: Diabetic nephropathy; PKD: Polycystic kidney disease, AKI: Acute kidney injury; T: Kidney transplantation; MN: Membranous nephropathy; KC: Kidney cancer. *sub class not available, ^#^class provided, ^¥^super class provided, ^§^not classified.

Metabolites	Sub Class	Kidney Disease						
		CKD	DN	PKD	AKI	T	MN	KC
allantoin	imidazoles	[122,129]	[139]	[120,152]		[147]		
quinolinic acid	pyridinecarboxylic acids & derivatives	[121]				[149]	[150]	[146]
2-hydroxyglutarate	short-chain hydroxy acids & derivatives		[15,139]	[154]			[150]	
2-oxoglutaric acid	gamma-keto acids & derivatives		[137]	[120,152]				[146]
aconitic acid	tricarboxylic acids & derivatives		[138]	[152]			[150]	
ADMA	amino acids, peptides & analogues	[131,136]		[152]	[123]			
carnitine	quaternary ammonium salts	[124,125]	[15]	[152]				
citrate	tricarboxylic acids & derivatives	[124,125]	[138,139]	[152,153,154]				
creatinine	amino acids, peptides & analogues	[121,125,127,132,133,135]		[152]	[123]			
hippuric acid	benzoic acids & derivatives	[122,128,132]	[15,137,139]	[120,152]				
kynurenic acid	quinoline carboxylic acids	[121]	[15]			[149]		
LysoPC (16:1)	Glycerophosphocholines	[135]	[139]		[123]			
malic acid	beta hydroxy acids & derivatives	[124]	[139]	[154]				
methionine	amino acids, peptides & analogues	[121,126]	[139]		[123]			
myo-inositol	alcohols & polyols	[127,132,134]	[139]	[152]		[149]		
threonic acid	carbohydrates & conjugates	[124]	[139]				[150]	
trimethylamine oxide	aminoxides	[132]		[152]		[149]		
tryptophan	indolyl carboxylic acids & derivatives	[121,133,135]	[15]		[123]			
uric acid	purines & purine derivatives	[121,122,126]	[139]	[120]				
valine	amino acids, peptides & analogues	[130,135]	[144]			[149]		
2-furoylglycine	amino acids, peptides & analogues	[121]						[146]
3-indoxyl sulfate	arylsulfates		[137]	[152]				
3-methylhistidine	amino acids, peptides & analogues	[132,133]		[152]				
4-pyridoxic acid	pyridinecarboxylic acids & derivatives	[121]	[139]					
4-hydroxymandelate	1-hydroxy-2-unsubstituted benzenoids	[126]				[149]		
acetylcarnitine	fatty acid esters			[152]	[123]			
alanine	amino acids, peptides & analogues	[121,134]	[139,144]					
arachidonic acid	fatty acids & conjugates	[122,124,129]	[139]					
arginine	amino acids, peptides & analogues	[122,126]			[123]			
citrulline	amino acids, peptides & analogues	[124,131]		[152]				
cytosine	pyrimidines & pyrimidine derivatives	[126]					[150]	
fructose	carbohydrates & conjugates		[139]					[146]
fumaric acid	dicarboxylic acids & derivatives		[140]	[154]				
gentisate	benzoic acids & derivatives					[149]		[146]
glucose	carbohydrates & conjugates	[134]	[139]					
glutamic acid	amino acids, peptides & analogues		[15]	[154]				
glutamine	amino acids, peptides & analogues	[121,130]		[154]				
glycine	amino acids, peptides & analogues	[125,130,134]		[152]				
homocysteine	amino acids, peptides & analogues	[133]			[123]			
hypoxanthine	purines & purine derivatives	[122]		[153]				
indole acetic acid	indolyl carboxylic acids & derivatives	[121]	[15]					
indoxyl sulfate	arylsulfates	[121,122,128,129]	[139]					
lactic acid	alpha hydroxy acids & derivatives	[134]	[139]					
leucine	amino acids, peptides & analogues	[131]				[149]		
lysine	amino acids, peptides & analogues	[121]	[139]					
Lyso PC (16:0)	Glycerophosphocholines	[135]			[123]			
Lyso PC (18:0)	Glycerophosphocholines	[135]			[123]			
Lyso PC (18:2)	Glycerophosphocholines	[135]			[123]			
Lyso PC (20:4)	*^§^	[135]	[139]					
N,N-dimethylglycine	amino acids, peptides & analogues	[132]		[152]				
ornithine	amino acids, peptides & analogues	[124]	[139]					
pantothenic acid	polyols	[121]		[152]				
phenylacetylglycine	amino acids, peptides & analogues	[129]	[137]					
phenylalanine	amino acids, peptides & analogues	[126,133]			[123]			
phosphate	non-metal phosphates	[124]	[139]					
pipecolate	amino acids, peptides & analogues			[152]		[149]		
proline	amino acids, peptides & analogues	[121,131]				[149]		
pseudouridine	nucleoside & nucleotide analogues*^#^	[121,127,132]	[15]					
pyroglutamic acid	amino acids, peptides & analogues		[139]		[123]			
taurine	organosulfonic acids & derivatives	[134]	[139]					
tetracosahexaenoate	fatty acids & conjugates	[122]	[139]					
threonine	amino acids, peptides & analogues	[126]	[139]					
trigonelline	alkaloids & derivatives*^¥^	[125,132]		[153]				
urea	ureas	[121]	[139]					
xanthosine	purine nucleosides*^#^	[121]				[149]		
xylitol	carbohydrates & conjugates					[149]	[150]	
